# The effects of irrigation volume to the heat generation during implant surgery

**DOI:** 10.4317/medoral.21880

**Published:** 2017-06-18

**Authors:** Alper Sindel, Ömür Dereci, Mükerrem Hatipoğlu, Ali Altay, Öznur Özalp, Adnan Öztürk

**Affiliations:** 1 DDS, PhD, Assistant Professor, Akdeniz University, Faculty of Dentistry, Department of Oral and Maxillofacial Surgery, Antalya, Turkey; 2 DDS, PhD, Assistant Professor, Eskişehir Osmangazi University, Faculty of Dentistry, Department of Oral and Maxillofacial Surgery, Eskişehir, Turkey; 3 DDS, PhD, Assistant Professor, Akdeniz University, Faculty of Dentistry, Department of Periodontology, Antalya, Turkey; 4 DDS, Research Assistant, Akdeniz University, Faculty of Dentistry, Department of Oral and Maxillofacial Surgery, Antalya, Turkey; 5 DDS, PhD, Professor, Erciyes University, Faculty of Dentistry, Department of Oral and Maxillofacial Surgery, Kayseri, Turkey

## Abstract

**Background:**

To evaluate the effects of the amount of irrigation on heat generated during implant site preparation.

**Material and Methods:**

Ten freshly dissected sheep mandibles were sectioned into 30 equal bone blocks and transferred into a heat-controlled water tank. Implant socket preparations were performed with four consecutive drills. Temperature measurements were performed with a thermocouple inserted into the bone immediately before the preparation and after the drilling using three different physiologic saline irrigation set-ups: 1- No irrigation, 2- 12 ml/min and 3- 30 ml/min irrigation volume. The temperature differences between three different irrigation set-ups for implant drills 1, 2, 3 and 4, and the temperature differences between the drills for three different irrigation set-ups were separately compared.

**Results:**

The temperature difference of no irrigation group was significantly higher than 12 ml/min and 30 ml/min groups for all four drills (*p*<0.05), whereas no statistically significant difference was found between 12 ml/min and 30 ml/min irrigation groups. (*P* >0.05) The temperature difference of drill 1 is significantly higher than drills 2, 3 and 4 for no irrigation group. (*P* <0.05) The temperature differences of drill 1, 2 and 3 were significantly higher than the temperature difference of drill 4 for 12 ml/min irrigation group. (*p*<0.05).

**Conclusions:**

The heat generated during drilling is not directly proportional to the coolant volume. Given that certain amount of irrigation is applied, implant sites can be prepared safely without the need for additional irrigation, which may result in reduced visibility of the surgical site and therefore a suboptimal surgery.

** Key words:**Dental implants, irrigation, heat generation, drilling.

## Introduction

Oral rehabilitation with osseointegrated dental implants has been extensively practiced worldwide with high success rates and accepted predictability (1). However, failures are encountered due to several reasons, including excessive surgical trauma during receptor implant site preparation and sequential mechanical and thermal damage (2). Preservation of healthy bone is an essential prerequisite for primary healing, which procures physiological osseointegration of dental implants (3). The characteristics of the surgery affect a series of events, which dictate the outcome and primary implant stability, which is arguably the most deterministic prerequisite of optimal osseous integration. Heat-induced tissue necrosis not only inhibits microcirculation of the bone and jeopardizes its regenerative capacity, but also endangers primary healing and osseointegration due to reduced initial implant stability (4,5). Atraumatic preparation and the avoidance of excess heat generation during the surgical procedure affect several phases of the peri-implant healing and overall treatment success.

Mechanical and thermal damage of the implant receptor bed during preparation can result in necrosis of the surrounding tissues. A temperature of 47 oC for 1 minute was documented to be the upper threshold for bony survival (6). However, the heat generated during surgery may exceed 70 oC under certain circumstances and have irreversible altering effects in the mechanical structure of the bone. The width of the necrotic zone that appears around the surgical defect is directly proportional to the magnitude of heat generated during the surgical procedure, which impairs the turnover in a number of ways, including hyperemia necrosis, osteolytic degeneration and increased osteoclastic activity (7).

It has been previously reported that the heat generated during implant receptor site preparation is affected by several factors, which include cortical bone thickness, drilling speed and depth, pressure applied during drilling, drilling pattern -intermittent or continuous- and the irrigation method applied (8). Among these, the role of different irrigation systems –conventional external vs. internal- has been subject to a number of studies, which compare the heat generated using these systems (9-11). However, the effects of different amounts of irrigation on heat generation during implant site preparation remain to be elucidated. In consideration of these premises, the aim of this in vitro study was to evaluate the effects of the presence and the amount of irrigation on heat generation during implant site preparation.

## Material and Methods

- Study Design

**Figure 1:**
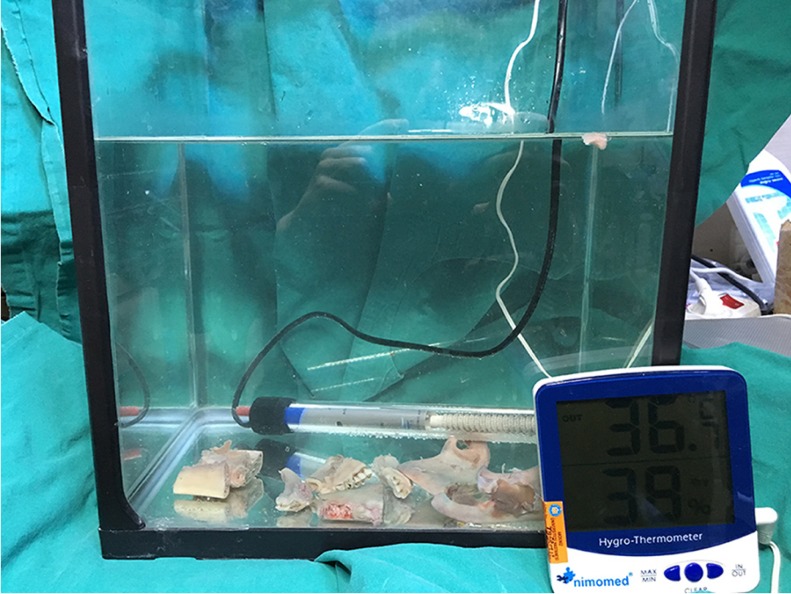
The dissected sheep mandible parts were transferred into a water tank of which the water temperature was adjusted as the body temperature of 35-37 degrees by a thermal controller.

**Figure 2:**
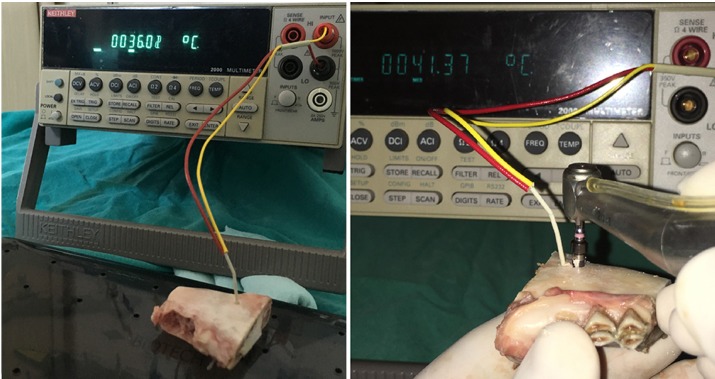
Thermocouple probe was inserted into the tissue at 3 mm distance to the drill insertion point.

**Figure 3:**
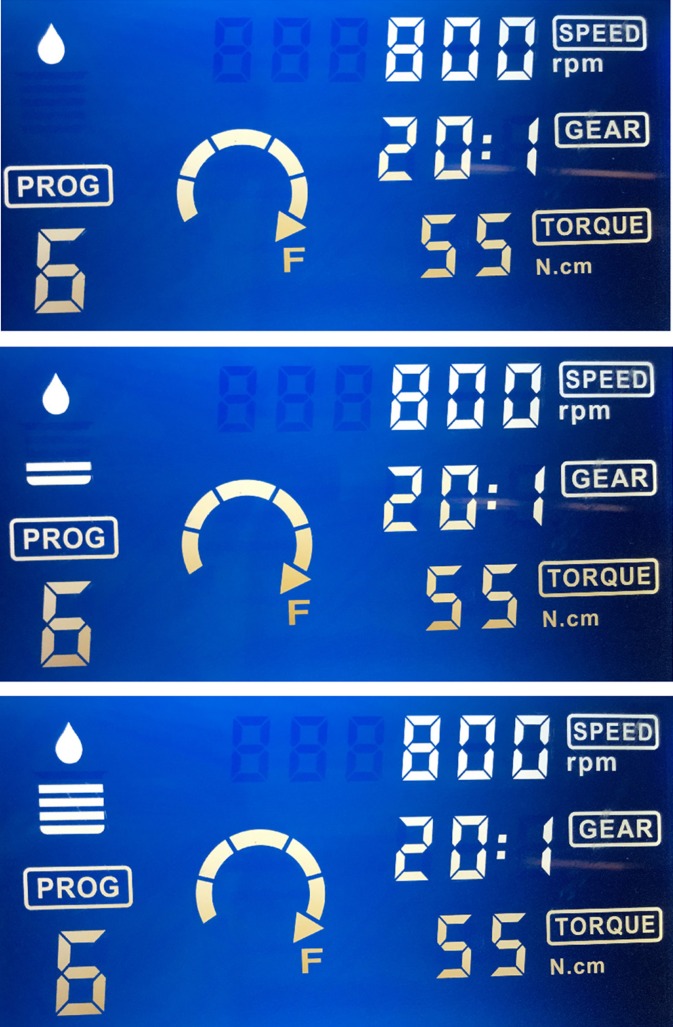
Three different physiologic saline irrigation set-ups of 1- No irrigation, 2- 12 ml/minutes irrigation volume and 3- 30 ml/minutes irrigation intensity were used.

The study protocol was approved by the Local Animal Research Ethics Commitee at Akdeniz University with approval number 557, and was performed in accordance with the ethical standards laid down in the 1964 Declaration of Helsinki and its later amendments. Ten freshly dissected sheep mandibles were sectioned into 30 equal parts on which holes were prepared at 3 mm distance to the drill insertion points for probe insertion. All specimens were obtained from the body portion of sheep mandibles. Bone blocks were then transferred into and kept in a water tank, the temperature of which was adjusted to the body temperature of 37 degrees oC by a thermal controller (Kems Angora) (Fig. 1). Bone blocks were removed from the water tank and measurement probe of a thermocouple device (Keitley 2000 Digital Multimeter, Keithley Instruments, Inc., USA) was inserted into the bone (Fig. 2). Implant socket preparations were performed with a sequence of four drills (drills 1, 2, 3, and 4) with a length of 14 mm. The diameters of the drills were 2.8 mm, 3.4 mm, 3.8 mm and 4.4 mm respectively. Temperature measurements were performed immediately before the implant site preparation and after the application of each drill using three different physiologic saline irrigation set-ups: 1- No irrigation, 2- 12 ml/min. and 3- 30 ml/min. irrigation volume (Fig. 3). Each irrigation method was used during drilling on ten bone blocks. The initial temperature before the drilling and the maximum temperatures reached during drilling were recorded. The mean temperature difference between the initial and the post-drilling measurements was calculated for each variable.

- Statistical Analysis

SPSS version 22.0 (IBM, Chicago, IL, USA) was used for statistical analyses. Shapiro-Wilk’s test (*p*<0.05) and visual inspection of their histograms, normal Q-Q plots and box plots showed that the exam scores were normally distributed for all heat measurements. *P* value was set at 0.05.

The temperature differences between three different irrigation set-ups for implant drills 1, 2, 3 and 4 were compared separately with ANOVA, while Sidak’s correction was performed for the post-hoc analysis. The temperature differences between drills 1, 2, 3 and 4 for three different irrigation set-ups were analyzed separately with repeated measures ANOVA, and Bonferroni correction was performed for the post-hoc analysis.

## Results

There were statistically significant differences between three irrigation set-ups for all four drills. (Ff1 = 115,871, Ff2 = 40,409, Ff3 = 34,746, Ff4 = 59,256, *p*<0.05) ( Table 1). Post-hoc analysis revealed that the temperature difference of no irrigation group was statistically higher than 12 ml/min and 30 ml/min groups for all four drills (*p*<0.05). There was no statistically significant difference between 12 ml/min and 30 ml/min irrigation groups (*p*>0.05).

**Table 1:**
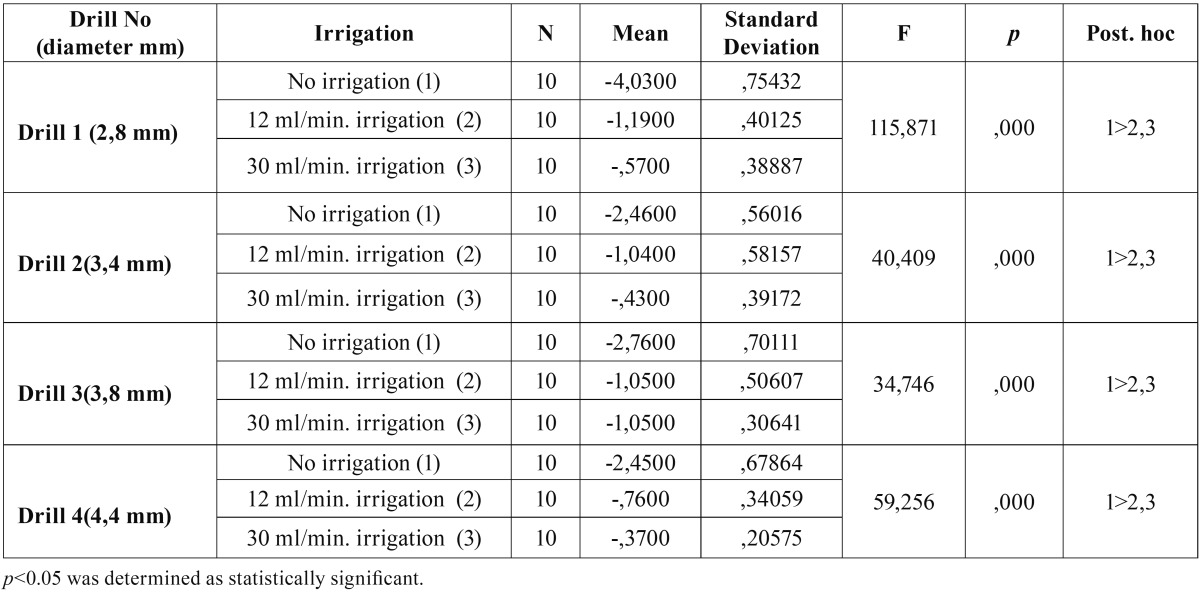
Comparison of the mean temperature differences between initial and maximum post-application temperatures for different irrigation procedures.

There was statistically significant difference between temperature differences of four drills for three different irrigation set-ups (*p*<0.05) ( Table 2). Post-hoc analysis revealed that the temperature difference of drill 1 is significantly higher than drill 2, 3 and 4 for no irrigation group (*p*<0.05), while the temperature differences of drills 1,2 and 3 were significantly higher than the temperature difference of drill 4 for 12 ml/min irrigation group.

**Table 2:**
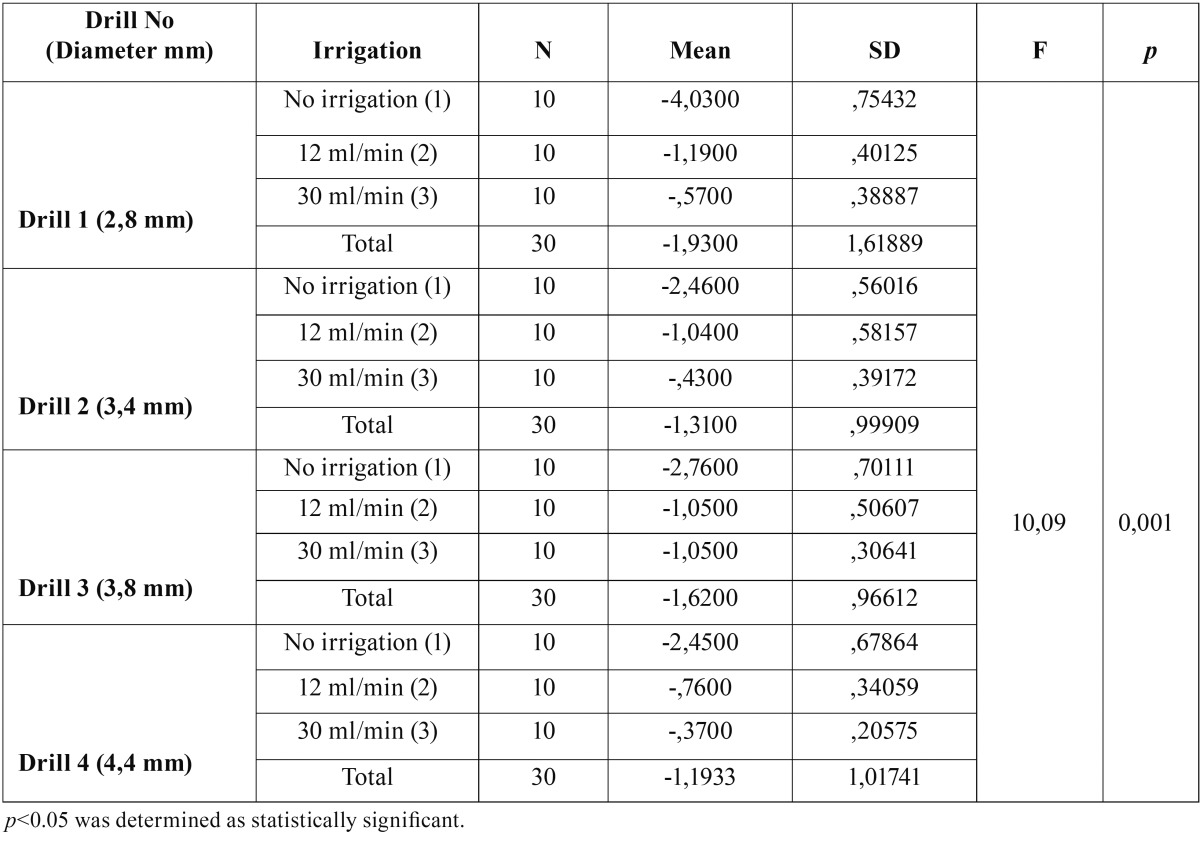
The comparison of the difference between initial and post-application maximum temperatures of 4 drills for 3 different irrigation set-ups.

## Discussion

Several implant or surgery related factors such as drill shape, flute design, drill speed, application force and structure of the host bone affect the heat generation during implant bed preparation (12,13). The success of the implant osseointegration is directly influenced by the heat generated during the preparation of the implant osteotomy site as temperatures higher than 47◦C in the bone tissue may result in osseous necrosis (6,7,13). To ensure a healthy osseointegration process, bone necrosis of the implant bed due to excessive heat should be avoided.

The structure, shape and geometric design of the implant drill are important factors that need to be taken into consideration during implant bed preparation. Several authors have previously reported that the drill type and shape may affect heat generation and implant bed osteotomy (9,10,12-15). Sannino *et al.* (14) reported that reduction in cutting surface in length can decrease the heat generation, and preliminary pilot drill use can decrease the frictional temperature rise independent from geometric design of the drill. Conversely, a number of authors have reported that the drill shape and type do not affect heat generation (10,15). Bullon *et al.* (10) have applied two different types of stainless steel drills on bovine ribs, and concluded that drill type and use do not have significant effects on heat generation, while irrigation was found to have a direct impact on temperature rise. Similarly, Koo *et al.* (15) reported that there was no significant difference in heat generation between different drill types that have different coatings, and irrigation was more important in controlling heat generation when compared to the drill type.

Drill speed is another important determinant that is to be considered in terms of heat generation in the implant drilling site. Although Kim *et al.* (16) previously reported that high speed drilling with high-torque and no irrigation does not significantly increase the temperature in the host bone, a review of the current literature reveals that heat generation increases in positive correlation with drill speed (17-19). A drill speed between 1000-2000 rpms was recommended to avoid excessive heat generation in cortical bone drilling (20). In the present study, a drill speed of 1000 rpm was used to avoid bias between drill groups during the implant bed preparation.

Cooling systems enable heat reduction in the implant bed by streaming the physiologic saline solution internally through the drill itself or externally onto the cutting part of the drill. It has been widely accepted that external cooling systems effectively prevent excessive heat generation. Boa *et al.* (21) found that the heat generated during drilling can be prevented and the thermal increase can be kept under the acceptable limit of 10°C using external cooling, whereas the increase may reach destructive limits when no irrigation is applied. Based on the hypothesis that the liquid flowing from the tip of the drill may more effectively reduce the heat compared to external cooling systems, the use of internal cooling systems have been previously suggested (22). However, studies comparing these two irrigation systems have failed to identify significant differences (11). Gehrke *et al.* (9) applied three groups of drill sequences with different diameters, and compared internal and external irrigation set-ups on bovine ribs. They reported that the system with both external and internal irrigation is more successful in decreasing the internal thermal heat in multiple conventional drill sequences even if the drill length increased. Likewise, a combined system of both internal and external irrigation is also recommended to maximize the irrigation effect, especially in deeper osteotomies in several studies (5,7,23).

In the current study, temperature measurements were performed after the application of each drill and the heat generated with no irrigation model was found to be significantly higher than other irrigation models as expected. However, there was no statistically significant difference between the low volume (12 ml/min) and the high volume (30 min/min) irrigation during implant site preparation, indicating that the increase in the coolant volume does not significantly decrease the heat generation in an external irriga-tion set-up. The initial drill with the smallest diameter (2,8 mm) revealed significantly increased heat generation when compared to other drills in no irrigation group. This finding supports the common idea that more heat is generated during first contact with the cortical bone, and when using a narrow drill that is smaller than 3 mm in diameter compared to drills with a diameter wider than 3 mm.

External irrigation is effective in reducing heat during implant bed preparation. Within the limitations of the current study among which are the low number of irrigation groups and use of a single thermocouple probe, it can be concluded that increasing the volume of the physiologic saline coolant does not correlatively provide increased heat reduction, and 12 ml/min coolant volume provides an effective heat reduction during implant bed preparation. In consideration of the findings of the present study, clinicians may consider limiting the amount of irrigation to a certain level during implant bed preparation, which may otherwise limit visibility of the surgical site when applied at excessive amounts. Further studies looking at different regions of osteotomy sites with the aid of multiple thermocouples and irrigation groups are required to gain an enhanced understanding of the effect of coolant volume on heat generation during implant bed preparation.
